# Case report: neonatal giant forehead hemangiopericytoma with a 5-year follow-up

**DOI:** 10.1097/MD.0000000000017888

**Published:** 2019-11-22

**Authors:** AiJun Peng, LiBing Zhang, Hai Zhao, LiangXue Zhou

**Affiliations:** a Department of Neurosurgery, West China Hospital, Sichuan University, Chengdu, Sichuan province; b Department of Pediatrics, Affiliated Hospital of Yangzhou University, Yangzhou, Jiangsu province, China.

**Keywords:** computed tomography angiography, hemangiopericytoma, infantile, surgery

## Abstract

**Rationale:**

Hemangiopericytoma (HPC) is a rare pediatric neoplasm with a high risk of bleeding, aggressive growth and high early relapse rates. Surgical excision remains the mainstream treatment, while the functions of chemotherapy and radiotherapy remain controversial. In particular, an infantile giant extracranial HPC located in the forehead has never been reported.

**Patient concerns:**

A 3-day-old girl was delivered normally with a giant tumor localized mainly in the right frontal region. The surface of the mass was filled with vascularity.

**Diagnosis:**

According to the results of imaging and pathological examinations, the diagnosis was HPC grade II.

**Interventions:**

Gross total resection of the tumor and the invading partial frontal bone followed by skin scalp reconstruction was carried out without any blood transfusion.

**Outcomes:**

No recurrence was identified during 5 years of follow-up. And better outcomes can be achieved without adjuvant therapy.

**Lessons:**

Multimodality imaging and a collaborative multidisciplinary approach are indispensable for the successful surgical management of infantile HPC, especially for giant tumors and their potential risk of life-threatening bleeding. Gross total resection is the optimal option for infantile HPC, and even without adjuvant therapy, it achieves better outcomes.

## 1 Introduction

Hemangiopericytoma (HPC), which was first described by Stout and Murray in 1942, is a hypervascular neoplasm originating from the pericytes of Zimmeman that is surrounded by capillary and postcapillary venules.^[1]^ Since 2002, cases of HPC have been grouped under the umbrella term ‘extrapleural solitary fibrous tumors (SFTs)’.^[2]^ Only after NAB2-STAT6 fusion was discovered using whole-exome sequencing was HPC reclassified from SFT to SFT combined with conventional HPC, resulting in a unique entity, SFT/HPC.^[3]^

Previous studies have shown that the incidence of head and neck HPC ranges from 4.5% to 10%^[2,4,5]^ and that intracranial HPC accounts for less than 1% of central nervous system (CNS) cases. Surgeries performed on infants remain a challenge due to surgery-related complications and outcomes. Here, we report a giant infantile head HPC patient who underwent gross total resection (GTR) of the tumor and had a beneficial outcome at a 5-year follow-up.

## 2 Case report

**Figure 1:**
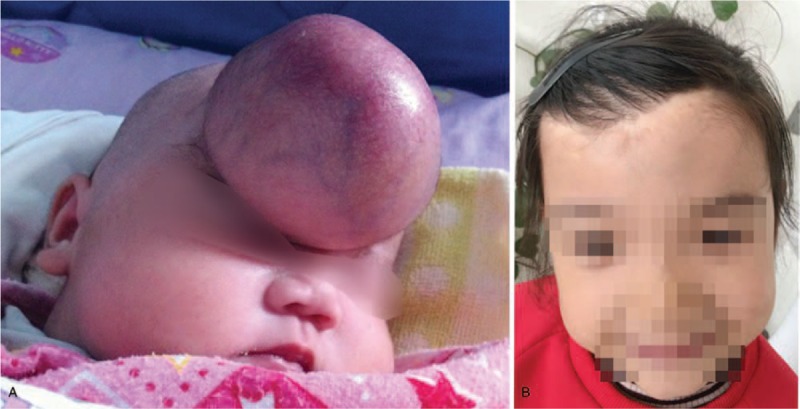
Pictures showing when the infant was admitted and 5 years later. (A) A giant tumor located on the forehead was full of many capillaries. (B) After a 5-year follow-up period, the wound could not be found without careful examination.

A 3-day-old girl was delivered normally with a giant tumor localized mainly in the right frontal region on July 22, 2013. On physical examination, the infant's weight was 4.7 kg, her height was 52 cm, and her head circumference was 39 cm. Additionally, a 9 × 7.1 × 5.1 cm soft, rounded and well-defined mass with a broad base occupied most of the forehead. The surface of the mass was filled with vascularity (Fig. 1A), and other clinical examinations showed that the mass had an intact status. A diagnosis of hemangioma was made at admission, and imaging studies were ordered.

**Figure 2:**
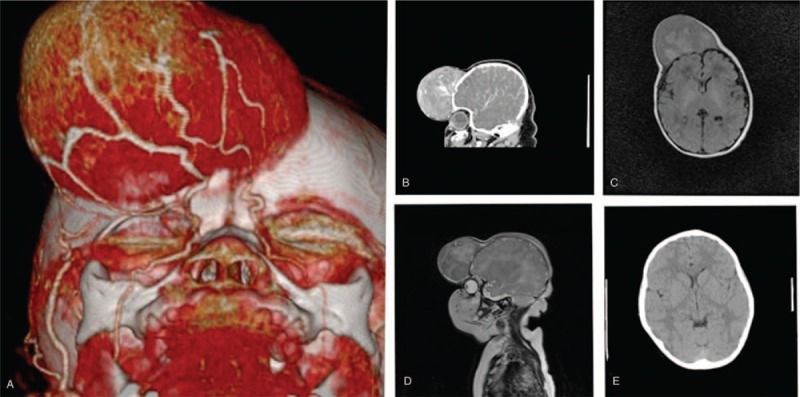
Multimodality imaging performed in this infant. (A) CTA demonstrated that the blood supply to the vascularized tumor originated mainly from the bilateral supratrochlear artery and partly from the distal part of the right superficial temporal artery. MRI showed a right extracranial mass with heterogeneous intensity on contrast-enhanced CT (B) and isointensity on T1-weighted (C) and T2-weighted images (D). There were no signs of recurrence or metastasis of the neoplasm on a CT scan (E).

A computed tomography (CT) scan revealed a large mass tumor located in the front of the head, and computed tomography angiography (CTA) demonstrated that the blood supply to the vascularized tumor originated mainly from the bilateral supratrochlear artery and partly from the distal part of the right superficial temporal artery (Fig. 2A). Moreover, magnetic resonance imaging (MRI) showed a right extracranial mass with heterogeneous intensity on contrast-enhanced CT (Fig. 2B) and isointensity on T1-weighted (Fig. 2C) and T2-weighted images (Fig. 2D).

**Figure 3:**
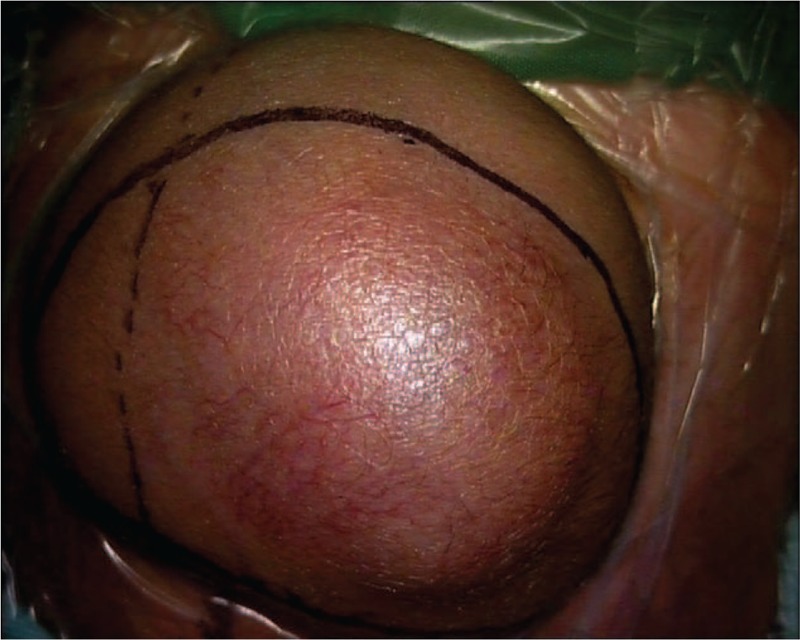
The incision of the infantile HPC. The incision was made along the black line, which could be used for skin reconstruction.

After a thorough preoperative evaluation with a clinical collaborative multidisciplinary team that included members of the neurosurgery, pediatric, anesthesiology and intensive care units and a discussion of perioperative management, the surgery strategy and any potentially risky situations that could arise during the operation, the surgery was performed. During the operation, a reasonable incision was designed (Fig. 3), and the main blood supply arteries were found and coagulated. Then, the tumor was completely removed successfully, and the eroded frontal bone was abraded with grinding drill of milling cutter. Following resection, the skin scalp was successfully reconstructed. A blood transfusion was not necessary due to a low rate of hemorrhage during the operation.

**Figure 4:**
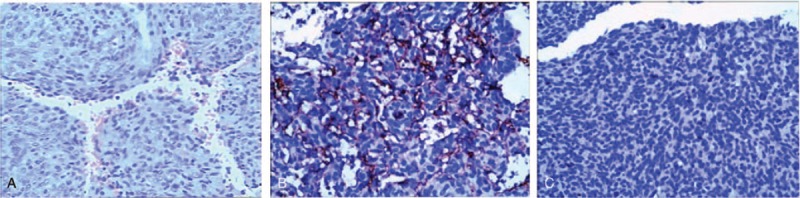
Histological examinations of the tumor. HE staining showing that the tumor cells had polymorphic nuclei that were round and oval-shaped and surrounded by typical staghorn-shaped blood vessels (A). Immunohistochemical staining showing that the cells were positive for CD34 (B). The rate of Ki-67 positivity was 5% (C). Original magnification × 200 (a) and × 400 (B and C).

Hematoxylin and eosin (HE) staining showed that the tumor cells had polymorphic nuclei that were round and oval-shaped, were surrounded by typical staghorn-shaped blood vessels (Fig. 4A), and showed prominent mitotic activity. Immunohistochemical staining showed that the cells were positive for CD34 (Fig. 4B) and negative for bcl-2, EMA, and S100 and that 5% of them were Ki-67-positive (Fig. 4C). This pathological profile was consistent with HPC grade II.

After the operation, the infant was sent to the intensive care unit to monitor her vital signs. There was no sign of bleeding in the surgery area on a CT performed on the 1st day after the operation, which revealed that a gross total resection of the tumor was achieved. Approximately one week later, the infant's wound became black. With careful wound nursing, she was discharged in good condition four weeks later. She did not receive chemotherapy or radiotherapy. No evidence of recurrence or metastasis was found during follow-ups performed 6 months after the operation and then annually and biannually once stability was confirmed. After a 5-year follow-up period, the wound could not be found without careful examination (Fig. 1B), and there were no signs of recurrence or metastasis of the neoplasm on a CT scan (Fig. 2E).

## 3 Discussion

HPCs originating from capillary pericytes are extremely rare pediatric tumors with signs and symptoms indicating aggressive behavior. This disease includes two distinct clinical types: infantile (occurring at < 1 year old) and adult (occurring at > 1 year old). Infantile HPC can occur anywhere in the body, including primary soft tissues, deeper structures and the lower extremities,^[6–8]^ but the most common site is the head and neck.^[9,10]^

We identified 38 cases of infantile extracranial HPC that were reported in the literature (Table 1). The locations of the tumors included the head^[11,12]^ (2 cases), tongue^[13,14]^ (2 cases), maxillary sinus^[15]^ (1 case), rhinopharynx^[16]^ (1 case), orbit^[17–19]^ (3 cases), neck^[12,20–24]^ (6 cases), trunk and back^[25–28]^ (7 cases), abdomen^[8,12,29–32]^ (5 cases), arms and legs^[12,26,33–36]^ (10 cases) and perianal region^[26]^ (1 case). The male to female ratio was 20 to 18 (Table 1). The maximum size of the lesions^[23]^ reported in the literature was 11×7 cm for a lesion located in the neck that achieved only a partial resection, while the smallest lesion was reported by Ragam et al.^[17]^ That lesion was 1.5×1.2×0.5 cm, was located in the orbit, achieved only a partial resection and was treated with chemotherapy. Suilt et al^[21]^ reported one small HPC (1.6 cm) with spontaneous regression under careful monitoring. The lesion in our case was located in the front region near the orbit and was larger than the previous lesions located in the head that were reported by Perez et al^[11,12]^ To the best of our knowledge, this is the first report to describe an infantile giant extracranial HPC located in the forehead.

**Table 1:**
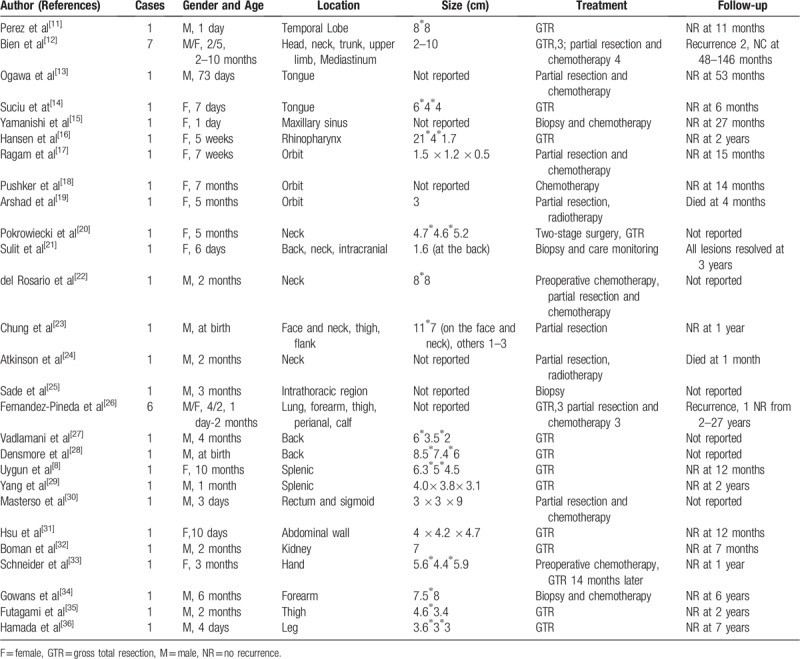
Summaries of the previously reported cases of extracranial infantile HPC.

The clinical symptoms of infantile HPC can include hemorrhage, compression of local structures, and even sudden death.^[4,5,20]^ Perez et al^[11]^ reported a neonate with HPC that caused hypovolemic shock due to bleeding in the temporal lobe; the patient underwent emergency surgery and was transfused with more blood. Ogawa et al^[13]^ described a case of infantile HPC located on the tongue that showed sudden bleeding and required a total of 32 units of red cell concentrate due to severe tumor bleeding. The giant tumor described in our report had an extremely high risk of bleeding. If it began to bleed, the infant's life would be at risk. Therefore, imaging examinations were very useful for quickly detecting the extent of the tumor, which helped the doctors select the best methods for treating the HPC during the perioperative period, even though there were no specific features for diagnosing infantile HPC^[37]^. In our case, both CT and MRI were very valuable for evaluating tumor invasiveness, whereas CTA was used for assessing the tumor blood supply and provided the surgeons with the ability to control bleeding during the operation.

HPCs represent a diversity of neoplasms ranging from well- to poorly differentiated tumors (low- to high-grade, respectively). They are divided into three grades based on histological subtype, including WHO grade I (SFT); WHO grade II (HPC), which contains more pump cells and has a “staghorn” vasculature; and WHO grade III (anaplastic HPC), which shows > 5 mitoses per high-power field (HPF). Although detecting the expression of CD34, bcl-2, CD99, CD31, S100, and EMA by immunohistochemical staining can be used to better differentiate SFT/HPC from meningioma and myofibroma, it is not generally effective in diagnosing SFT/HPC.^[38]^ Han et al^[39]^ showed that 40% of metastatic and 85% of nonmetastatic tumors had positive CD34 immunostaining. Bertero et al^[40]^ demonstrated that in HPC, the mean rate of CD34 expression was 64%, while the rate for Bcl-2 was 63%. Our case showed typical pathological manifestations, including more tumor cells and a “staghorn” vasculature on hematoxylin and eosin staining (HE), and was CD34-positive for immunostaining.

In terms of treatment for infantile HPC, we identified 19 cases previously reported in the literature that were treated with complete resection; these included two cases^[12,33]^ treated with preoperative chemotherapy that achieved GTR of the tumor and one case^[20]^ treated with two-stage surgery including external carotid artery sacrifice without follow-up. Fourteen cases were treated with partial resection and chemotherapy, 2 with biopsy and chemotherapy, and another 2 with only biopsy. Additionally, Pushker et al^[18]^ reported one lesion in the orbit that was treated with only chemotherapy and was followed up for 14 months without recurrence. Two cases reported by Atkinson et al^[19,24]^ were tumors located in the orbit that were treated by partial resection and radiotherapy, but both infants eventually died at 1 and 4 months old. In our case, we devised a comprehensive and scientific preoperative plan and carefully operated to resect the tumor and adjacent affected frontal bone, without any adjuvant therapy, the infant showed no recurrence at the most recent follow-up.

According to the literature, the mainstay treatment for HPC is total tumor resection whenever possible.^[20,41]^ The risk of surgery-related morbidity and mortality in infantile HPCs increases with the amount of bleeding, and this may be the greatest barrier to complete resection. CTA or digital subtraction angiography (DSA) can be used to identify the main supplying blood arteries and is an indispensable adjuvant examination approach in HPCs. Hanak et al^[42]^ reported achieving GTR in 59% of adult HPCs with preoperative embolization, which was negatively correlated with intraoperative blood loss. It was only the case reported by Ogawa,^[13]^ which involved severe bleeding from a tumor in the tongue, in which embolization of the lingual artery with iodized oil was performed before partial resection. Chemotherapy was also considered in that case.

Although chemotherapy and radiotherapy can reduce local recurrence and metastasis, their functions need to be prospectively validated. Some studies^[20,43]^ have suggested that there is no need for chemotherapy. Bien et al^[12]^ showed that in cases of infantile-type HPC with unresectable, life-threatening tumors, chemotherapy could produce a favorable outcome, while macrotumors or unresectable adult-type HPCs should be considered for chemotherapy and radiotherapy. A systematic review by Spina et al^[44]^ revealed that Gamma Knife radiosurgery (KGS) could be a feasible and effective therapy for HPCs.

Overall, the outcomes of infantile HPC are different from those found in adult HPC due to differences in treatments and tumor locations. In the literature, the longest follow-up (2–27 years) for infantile HPC was reported by Fernandez, et al^[26]^ In their report, the tumors were located in the lung, forearm, thigh, perianal area and calf, but they did not report on tumor size. Perez et al^[11]^ followed up a case for 11 months after resection of a temporal lobe HPC. The follow-up time was 90 months after secondary surgery and chemotherapy for an HPC located in the head that was reported by Bien et al^[12]^ In our case, although the tumor was giant and had a risk of hemorrhage that threatened the infant's life, with a collaborative multidisciplinary approach, we determined that a complete resection without adjuvant therapy would achieve a better outcome.

In summary, multimodality imaging and a collaborative multidisciplinary approach are indispensable for the successful surgical management of infantile HPC. GTR, even without adjuvant therapy, is the better option for infantile HPC when there is a potential risk of threat to the infant's life. However, a longer follow-up period is essential due to the aggressive behavior of HPCs. We hope that this case report may be helpful in the efficient management of infantile HPC, especially in giant tumors with their potential risk of life-threatening bleeding.

## Author contributions

**Conceptualization:** AiJun Peng, LiBing Zhang

**Methodology:** LiangXue Zhou, AiJun Peng, LiBing Zhang

**Conceptualization:** AiJun Peng, LiBing Zhang, LiangXue Zhou.

**Formal analysis:** Hai Zhao, LiangXue Zhou.

**Methodology:** LiBing Zhang, LiangXue Zhou.

**Supervision:** LiangXue Zhou.

**Writing – original draft:** AiJun Peng, LiBing Zhang.

**Writing – review & editing:** Hai Zhao, LiangXue Zhou.
